# The Use of Registries to Improve Cancer Treatment: A National Database for Patients Treated with Interleukin-2 (IL-2)

**DOI:** 10.3390/jpm4010052

**Published:** 2014-03-07

**Authors:** Howard L. Kaufman, Michael K. Wong, Gregory A. Daniels, David F. McDermott, Sandra Aung, James N. Lowder, Michael A. Morse

**Affiliations:** 1 Department of Surgery, Rutgers Cancer Center Institute of New Jersey, 195 Little Albany Street, Room 2007, New Brunswick, NJ 08901, USA; 2 Department of Medicine, University of Southern California, 1441 Eastlake Avenue, Suite 3455, Los Angeles, CA 90033, USA; E-Mail: mike.wong@med.usc.edu; 3 Moores Cancer Center, University of California San Diego, 3855 Health Sciences Drive, La Jolla, CA 92093, USA; E-Mail: gdaniels@ucsd.edu; 4 Beth Israel Hospital Deaconess Medical Center, Masco Building, 375 Longwood Avenue, Boston, MA 02215, USA; E-Mail: dmcdermo@bidmc.harvard.edu; 5 Prometheus Laboratories Inc., 9410 Carroll Park Drive, San Diego, CA 92121, USA; E-Mails: sandra.aung@prometheuslabs.com (S.A.); jlowder@prometheuslabs.com (J.N.L.); 6 Duke University Medical Center, 10 Bryan Searle Drive, Mudd Building, Rm 437, Durham, NC 27710, USA; E-Mail: Michael.morse@duke.edu

**Keywords:** oncology, cancer, registry, immunotherapy, interleukin-2, melanoma, renal cell carcinoma

## Abstract

Registries evaluating un-randomized patients have provided valuable information with respect to a therapy’s utility, treatment practices, and evolution over time. While immunotherapy for cancer has been around for more than three decades, data collection in the form of a registry has not been undertaken. The authors believe that establishing a registry to study HD IL-2 immunotherapy, which has been the only systemic therapy producing long term unmaintained remissions for advanced kidney cancer and melanoma for over 20 years, will be an important resource in understanding the impact of immunotherapy with HD IL-2 in a rapidly changing therapeutic environment. Optimizing administration and improving selection of appropriate patients likely to benefit from HD IL-2 immunotherapy are two of many benefits to be derived from this endeavor.

## 1. Introduction

The best insights into high complexity therapy are gleaned through careful observation over time. Although randomized Phase III clinical trials are considered the gold standard, they often remain unpublished; they answer a very specific question and quickly become less relevant as medical practice evolves [1,2,3]. On the other hand, registries are designed to collect data based on real-world experiences over a long period of time and are designed with the sole purpose of observing and collecting data for publication. More importantly, registries may answer questions that a clinical trial could never address. A registry that has transformed medical practice is the International Bone Marrow Transplant Registry (IBMTR) [4]. An example of an important observation impenetrable to a Phase III randomized trial is the comparison of matched sibling donors to HLA matched unrelated donors. Other questions in transplantation such as preparative regimens and source of stem cells queried by randomized trials have suffered from conflicting outcomes, underpowering and the confounding of different management practices at individual sites. 

The development and clinical implementation of high-dose interleukin-2 (IL-2) (HD IL-2) therapy has many similarities with the development of HSCT. HD IL-2, like HSCT, is administered as in-patient therapy in specialized institutions with experienced and trained staff who manage patients through moderate to severe complications and/or toxicities all of which ultimately subside. Methods of managing adverse events are intensive and variable from site to site. Finally, the most important endpoint for both therapies is long term survival measured in decades.

The authors believe that establishing a registry to study HD IL-2 immunotherapy modeled on the IBMT registry will be an important resource in treating and managing patients with mRCC and mM, potentially increase our understanding of the underlying therapeutic mechanisms and possibly lead to new insights into the selection of appropriate patients likely to benefit from HD IL-2 immunotherapy. Unlike projects such as National Cancer Database (NCDB) and Surveillance, Epidemiology, and End Results (SEER) program which is a large database that collects patient demographics, death rates, and focuses on baseline characteristics, an actively managed registry, combined with the judicious analysis of its data can change treatment paradigms and positively impact patient morbidity, quality of life and survival. 

## 2. IL-2 Therapy over the Past Two Decades

In 1992, aldesleukin, a human-recombinant interleukin-2 product, was approved for the treatment of mRCC. The approval for aldesleukin in mRCC was based on seven Phase II clinical trials in 255 patients [5]. An objective response was seen in 37 (15%) of patients with 17 (7%) being complete responders and 20 (8%) being partial responders. The responses were usually rapid and were seen in patients with high tumor burdens and multiple metastatic sites. Similar data in patients with metastatic malignant melanoma (mM) were the basis for approval in 1998 for the treatment of that indication. Two hundred and seventy patients with mM were enrolled into eight clinical trials. Objective responses were seen in 43 (16%) patients with a complete response in 17 (6%) and a partial response in 26 (10%) patients [6]. As with mRCC patients, the responses were seen in lung, liver, lymph node, soft tissue, adrenal and subcutaneous sites. Most importantly, in both diseases these responses have proven to be durable. Augmented in some cases by surgical excision of the residual disease, but otherwise without further therapy more than 10% of patients remain alive and disease free after HD IL-2. This plateau or tail of the survival curve is a unique feature of HD IL-2 therapy and perhaps another immunotherapy, ipilimumab, which may represent cure of a metastatic solid tumor. The median DFS of complete responders from HD IL-2 exceeds 12 years in both mM and mRCC [7,8].

The FDA approved dose and schedule of HD IL-2 for mRCC and mM was defined as 600,000 IU/kg (0.037 mg/kg)/dose administered every 8 h by a 15 min intravenous infusion for a maximum of 14 doses. Following 9 days of rest, the schedule is repeated for up to another 14 doses for a maximum of 28 doses per course, as tolerated. However, the number of doses per cycle, the intervals between doses and cycles varies considerably from site to site. This high-dose bolus administration schedule has become the standard of care and other lower dose and continuous infusion schedules have not shown similar therapeutic benefits. In a study of 189 patients with mRCC it was shown that higher response rates and a more durable response were obtained when patients were given high dose IL-2 regimens than patients treated with lower dose regimens, ORRs 21% compared to 11%, respectively [9]. In a randomized Phase III trial of HD IL-2 *versus* subcutaneous LD IL-2 and interferon, ORR was 23.2% compared to 9.9%, respectively [10]. Thus lower dose regimens of IL-2 yielded significantly fewer and less durable responses than those obtained with the approved regimen, which is referred to as HD IL-2. Studies of HD IL-2 in mM mirrored those seen in mRCC with respect to response rate and durability of response [6]. HD IL-2 in the modern era has taught us that ORRs for mRCC is higher than historical response rates of 15% in 1995 [5]. In 2008, a single institution study by the NCI, reported an ORR of 20% for patients treated between 1986 and 2006 (*n* = 259) [11]. In 2010, the Cytokine Working Group (CWG) reported 25% ORR (*n* = 120) [12]. 

Although HD IL-2 is the only available treatment for both mM and mRCC that results in durable long-term responses greater than 10+ years in patients with stage IV mRCC and mM, the frequency of complete response is generally less than 10%. Researchers have conducted studies using IL-2 in combination with a variety of other immunotherapeutic agents to try to develop combinatorial or synergistic outcomes [13,14]. Interferon-α is an immunomodulatory cytokine which has been shown to be capable of producing tumor regression in preclinical models [15,16]. Because both IL-2 and IFN-α can induce cytotoxicity mediated by NK cells the hypothesis for combination therapy was advanced and several clinical trials were conducted. A CWG sponsored study, compared HD IL-2 with low dose (LD) IL-2 and INF-α in 192 patients with mRCC, only HD IL-2 had a 3 y durable response rate (7% *versus* 0%) [10]. The response rate was also greater in the group treated with HD IL-2 (23.2%) compared to the LD IL-2 + IFN-α group (9.9%). The authors concluded that this randomized Phase III trial provided additional evidence that HD IL-2 should remain the preferred therapy for selected patients with metastatic renal cell carcinoma [10]. 

Researchers have also developed vaccines directed at eliciting an immune response to melanoma tumors. Early *in-vitro* studies demonstrated that vaccination with the peptide gp100 resulted in high levels of circulating T cells capable of recognizing and killing melanoma cells [17]. Such results suggested that activation of these T cells by a vaccine in combination with cytokines such as IL-2 could be synergistic. A small study in 31 patients reported that immunization with the peptide vaccine followed by HD IL-2 led to an objective clinical response in 13 (42%) of the patients [18]. In order to confirm these results, a group of investigators conducted a phase 3 randomized, multicenter study in 185 patients with mM comparing IL-2 alone *versus* gp100 once per cycle followed by IL-2. The vaccine + IL-2 group as compared with the IL-2 alone group had a significant improvement in objective clinical response (16% *versus* 6%, *p* = 0.03) as well as longer progression free survival and a trend toward improved median overall survival [19]. 

CTLA4 is an antigen expressed on the surface of T cells and it is responsible for transmitting an inhibitory signal to T cells. Inhibiting this action, by administration of drugs such as ipilimumab, results in objective clinical responses in patients with mM [20]. A study at the NCI examined the effects of simultaneously administering HD IL-2 and ipilimumab to determine if there was any additional benefit. Although the study was not a comparative one, the results suggest that the combination had an increased complete response rate when compared to either HD IL-2 or ipilimumab alone in studies conducted at the NCI. A subsequent evaluation of patients receiving both drugs showed that 17% were in complete remission at a median follow up of 84 months post therapy [21]. Clinical trials evaluating the use of HD IL-2 and ipilimumab are underway.

Understanding the genetic components which drive the unchecked proliferation, lack of cell death and immune invisibility which characterize cancers affords another opportunity for treatment. The MAP kinase pathway has been shown to play a key role in cell growth and mutations of the BRAF gene are common in several types of cancer. Approximately half of all melanomas carry the BRAF V600E mutation [22]. When compared to dacarbazine in mM patients with the BRAF mutation, the BRAF inhibitor vemurafenib significantly reduced the number of deaths, improved progression free survival and had a best overall response rate of 48.4% compared to 5.5% with dacarbazine [23]. Progression free survival improved to a median of 5.3 months from 1.6 months (Hazard ratio, 0.26). Clinical trials to evaluate vemurafenib in combination with IL-2 are ongoing.

Autologous transfer of tumor infiltrating lymphocytes (TIL) can mediate durable regression of metastatic melanoma when administered with IL-2 therapy in patients that have been lymphodepleted [24]. Twenty of the 93 patients (22%) achieved a complete tumor regression, and 19 of the 20 remain disease free for more than 5 years [24]. However, not all patients with metastatic melanoma can receive this treatment approach. The metastatic nodule must be at least 2-cm in diameter and only half of the resections grow cells suitable for infusion. Current efforts are aimed at developing simpler and faster methods to grow TIL for adoptive immunotherapy [25].

## 3. Predictive Biomarkers of IL-2 Clinical Response

Although HD IL-2 clearly benefits some patients, they are in the minority. The ability to predict those patients most likely to respond to the drug would have significant value, sparing non-responding individuals the toxicity of treatment. Efforts to discover and validate predictive biomarkers have been a focus for years and thus far have fallen short. High serum levels of vascular endothelial growth factor (VEGF) and fibronectin were identified as independent predictors of non-response in patient with mM correlating with a lack of clinical response and decreased overall survival [26]. In patients with mRCC, the level of carbonic anhydrase IX-G250MN (CAIX) expression in tissue samples was promising as a potential predictor of biological response to IL-2 [27]. Clear cell histology of the primary tumor was associated with a higher response rate to HD IL-2 when compared with patients with non-clear cell histology [28]. Additional pathologic “good predictive features” in the clear cell group helped specify which patients within that particular group were more likely to respond to therapy [28]. These features were combined with CAIX expression to create good and poor risk groups [29]. However, when these retrospective observations were tested in a prospective trial, only the clear cell histology remained predictive of outcomes [12]. Additionally, PD-L1 expression on mRCC correlated with a doubling of ORRs in response to IL-2, going from 25% to 50% in the PD-L1 subgroup analysis [30]. Data has been collected from large cohorts of human cancers, demonstrating the impact of immune classification within the tumor as a prognostic [31,32,33,34,35]. In melanoma, high numbers of CD8+ T cell infiltrates within metastatic melanoma correlated with prolonged survival [36]. Introduction of this parameter as a biomarker to classify tumors, Immunoscore, as part of a routine prognostic assessment of tumors has been an on-going initiative [37]. 

## 4. Managing IL-2 Toxicity

A major barrier to IL-2 administration has been the development of cytokine-related toxicity which has concerned referring physicians. HD IL-2 induces a capillary leak syndrome that is characterized by hypotension, tachycardia and peripheral edema. HD IL-2 has also been associated with constitutional symptoms, such as fever and chills, nausea, vomiting, diarrhea, renal insufficiency, elevation of the hepatic enzymes, pruritus, myocarditis and cardiac dysrhythmias, confusion, hallucinations, thrombocytopenia, leucopenia, hypocalcemia, hypomagnesemia, autoimmune thyroiditis and vitiligo [38]. The entire syndrome is acute and is managed by experienced physicians and nurses in a hospital setting where appropriate monitoring and support are available. The side effects are typically reversible with cessation of treatment except for occasional thyroiditis and vitiligo, which may persist and have been associated with strong anti-tumor immunity and good clinical response in melanoma [39]. There is evidence that the incidence and severity of adverse events have decreased as health care professionals have become more competent in recognizing and managing the side effects during treatment and understanding when to delay or discontinue dosing of IL-2 [40]. Opportunities for training in immunotherapy administration for physicians and nurses in IL-2 have been limited. Thus, although the clinical benefits of IL-2 have been well established, patient access to treatment has been problematic due to a limited number of sites skilled in IL-2 administration and concerns about toxicity related to IL-2 treatment. A major goal of the registry is to survey current practices for the safe and efficient treatment of patients with HD IL-2 and generate modern data on its toxicity and outcomes. 

## 5. Interleukin-2 Registry

It is unknown how patient selection, specific dosing and toxicity management practices influence outcomes. In addition, the rapid introduction of new approved agents into the therapy choices for mRCC and mM continuously create new questions about sequencing agents which can’t be evaluated in a timely manner. Concerns of treatment related toxicities are a major barrier to IL-2 administration. Nonsystematic reviews show that HD IL-2 therapy is safe; however practice protocols have evolved in relative isolation at each of over 60 HD IL-2 centers in the US. In order to capture these current practices and generate modern data on toxicities and outcomes a HD IL-2 registry was initiated called PROCLAIM^SM^ (Proleukin^®^ Observational Registry to Evaluate the Treatment Patterns and Clinical Response in Malignancy). PROCLAIM is a US-based multi-center study designed to establish a high quality observational database of real-world clinical data on HD IL-2 when it is used to treat patients with mM, mRCC or other metastatic malignancies, whether as sole therapy or when it is used in conjunction with other agents. The registry opened enrollment on September 2011 and began collecting data from two cohorts, retrospective and prospective. The retrospective cohort, consisting of 267 mRCC and mM patients, enrolled patients between January, 2007 and February, 2012. The prospective cohort started enrolling patients September 2011. The goals of the registry are listed in Table 1. Of the 60 or more experienced HD IL-2 centers in North America, 35 sites comprise the participants in the registry (Table 2). These sites are representative of the total universe of sites, community and academic, with variation in the proportion of mRCC and mM patients, total number of patients treated per year, and geographic location.

**Table 1:** Goals of the Proleukin^®^ Observational Registry to Evaluate the Treatment Patterns and Clinical Response in Malignancy (PROCLAIM) registry.

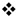 Provide information regarding IL-2 and its prospective use
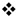 Compare the difference in administration approaches for their respective effect on outcomes
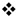 Validate efficacy of HD IL-2 on response and survival in the treatment of malignant diseases
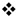 Identify patient and site specific prognostic factors
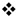 Study and potentially guide the emergence of new therapeutic options in the immunological armamentarium

**Figure 1:**
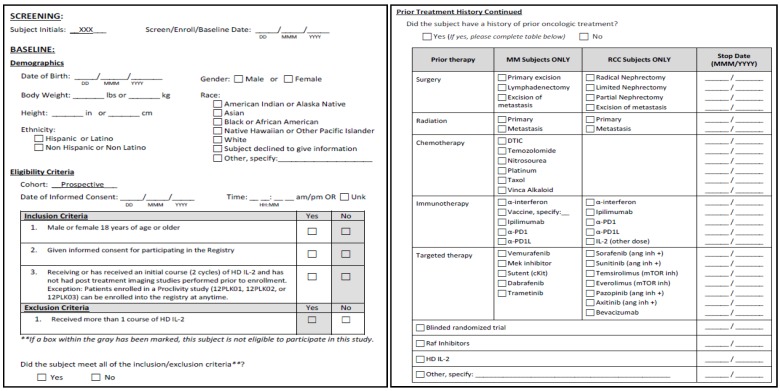
Example of data collected on the electronic database collection (EDC) system.

**Figure 2:**
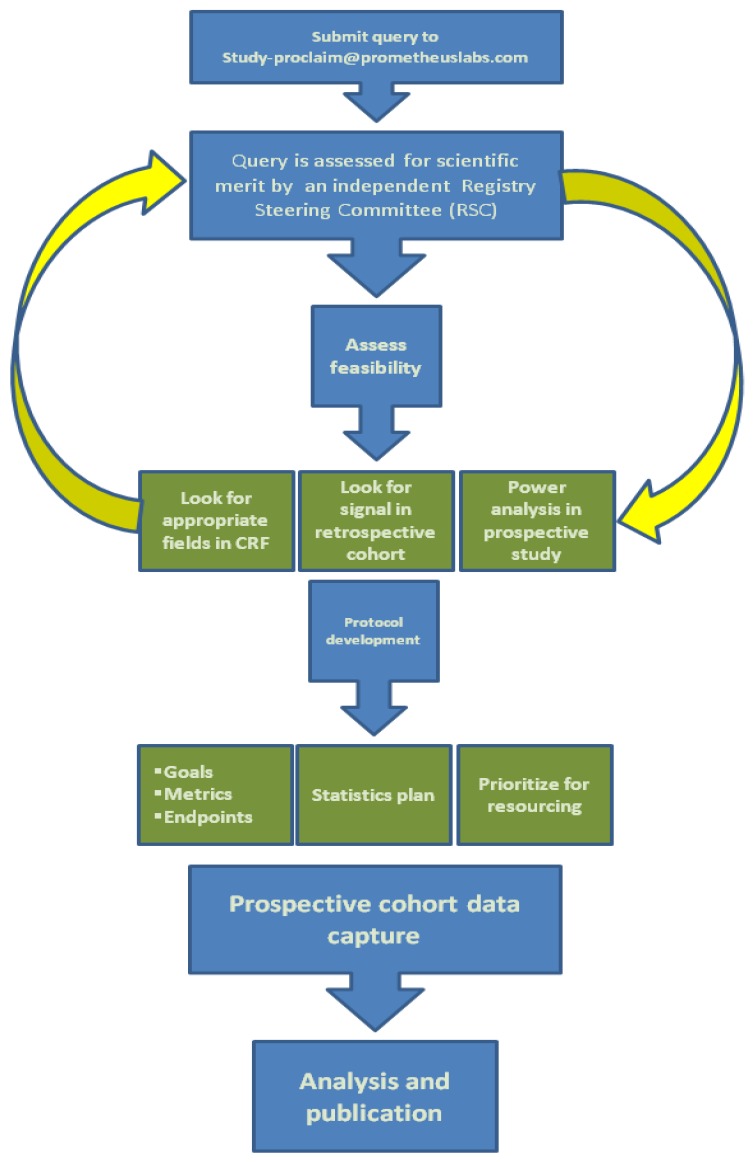
Query process for the PROCLAIM registry.

A Registry Steering Committee (RSC) is in place and provides scientific guidance and acts on behalf of the Sponsor (Prometheus Laboratories, Inc., San Diego, CA, USA) and key stakeholder groups. A charter has been created to define the composition and membership, development of the protocol, publications and yearly reports, as well as the approval process for important RSC decisions. The protocol for the study defines that standard clinical data is collected from each patient through an electronic data collection (EDC) system. The data collected will include demographics, prior treatment, dosing, reasons for stopping HD IL-2, laboratory values, clinical response and survival. Exemplary screens are shown in Figure 1. The database created will provide a source of information designed to answer a wide array of research questions. The ability to query the prospective data will be available to investigators, including those not part of the Registry network via a query process which is shown in Figure 2. It is the responsibility of the RSC to review and approve any such requests.

**Table 2:** Current PROCLAIM registry sites and principal investigators.

#	State	Registry Site	Principle Investigator
1	AZ	The University of Arizona Cancer Center	Joanne Jeter MD
2	CA	University of California San Diego	Gregory Daniels MD PhD
3	CA	USC Norris Cancer Center	Michael Wong MD PhD
4	CO	University of Colorado Cancer Center	Rene Gonzalez MD
5	FL	H. Lee Moffitt Cancer Center and Research Institute	Mayer Fishman MD PhD
6	FL	Mount Sinai Medical Center Comprehensive Cancer Center	Jose Lutzky MD
7	GA	Emory University Winship Cancer Institute	David Lawson MD
8	IA	University of Iowa Hospitals and Clinics	Mohammed Milhem MD
9	IL	Loyola University Medical Center	Joseph Clark MD
10	IL	Oncology Specialists, SC	Sigrun Hallmeyer MD
11	IL	Rush University Medical Center	Howard Kaufman MD
12	IN	Indiana University Simon Cancer Center	Theodore Logan MD
13	KS	University of Kansas Hospital	Peter Van Veldhuizen MD
14	LA	The Baton Rouge Clinic, AMC	Gerald Miletello MD
15	MA	Beth Israel Deaconess Medical Center	David McDermott MD
16	MI	Barbara Ann Karmanos Cancer Institute	Ulka Vaishampayan MD
17	MN	University of Minnesota Masonic Cancer Center	Venkatesh Rudrapatna MD
18	MO	Saint Louis University	John Richart MD
19	NC	Blumenthal Cancer Center	Asim Amin MD PhD
20	NC	Duke University Medical Center	Michael Morse MD
21	NC	Wake Forest University Baptist Medical Center	John Stewart IV MD
22	NE	Midwest Cancer Center—Legacy	Ralph Hauke MD
23	NH	Dartmouth Hitchcock Medical Center	Marc Emstoff MD
24	NJ	Hackensack University Medical Center	Robert Alter MD
25	NY	Saint Luke’s-Roosevelt Hospital Center	Seth Cohen MD
26	OH	University Hospitals Siedman Cancer Center	Henry Koon MD
27	OR	Providence Portland Medical Center	Brendan Curti MD
28	PA	Hillman Cancer Research Pavilion, Div. of Medical Oncology	John Kirkwood MD
29	PA	Saint Luke’s Hospital and Health Network	Sanjiv Agarwala MD
30	TX	MD Anderson Cancer Center	Sapna Patel MD
31	UT	University of Utah School of Medicine	Neeraj Agarwal, MD
32	OH	The Christ Hospital Cancer Center	Philip Leming, MD
33	MI	University of Michigan	Christopher Lao, MD
34	MD	John Hopkins University School of Medicine	William Sharfman, MD
35	NY	Columbia University Medical Center	Bret Taback, MD

Prior to data collection or enrollment of patients at any site, the protocol and its associated consent form must be approved by the respective IRB. Any patient undergoing HD IL-2 treatment at a participating clinical trial site will be eligible for inclusion into the Registry provided they sign an IRB-approved informed consent document prior to treatment for the prospective cohort. Ideally, all patients at participating sites will be offered the opportunity to enroll in the trial. Data is collected at baseline and at typical milestones during the treatment of HD IL-2. Data is uniformly collected for every cycle, at the standard tumor assessment after each HD IL-2 course (2 cycles) and at a standard interval follow up (approximately every 6 months). Principle Investigator determined tumor responses to HD-IL-2 are categorized using either WHO or RECIST criteria, depending on individual physician and site standards. Patient survival data will also be captured. The PROCLAIM Registry will generate an annual report tracking the status of the database and providing summary tables on the current use of HD IL-2 and outcomes. 

Specific fields not found in databases such as SEER or NCDB which are specific to HD IL-2 administration are included in PROCLAIM in order to understand administration variables. These unique features include specifics about dosing, reasons for stopping dosing and maximal excursions of pertinent laboratory values, as well as standard institutional order sets. In order to test the EDC and the appropriateness of data fields, a pilot study collected retrospective data at a subset of sites. This endeavor entered data into the EDC from the medical charts of 269 subjects previously treated with HD IL-2 for the treatment of malignancy. From 1 to 49 consecutively treated patients were enrolled at each of 13 heterogeneous sites. The data from this preliminary database will be used to validate and guide refinements of the prospective database. It will also be used to test hypotheses, in terms of the presence of a signal, powering a prospective study and determining whether a question can be adequately answered by the existing features of the database. Because this is an observational database, there are several inherent limitations. First physician determined assessment of disease progression will have wide variations. In the retrospective study, data could have been entered on only those patients that performed well. We did not directly capture referral patterns at academic compared to community centers. As the data entry grows prospectively, we will be able to elucidate key differences between drug sequences that have not been tested in randomized clinical trials.

The lack of a broad understanding among the oncology community was recently cited as a major barrier to the integration of tumor immunotherapy into the cancer therapeutic armamentarium [41]. The durable therapeutic responses without further therapy achieved by HD IL-2 in many mRCC and mM patients may not be appreciated by most physicians. Considerable differences in the management of patients exist among sites administering HD IL-2 with minimal information regarding their impact optimizing outcomes. The PROCLAIM^SM^ Registry will provide a national resource for the collection of IL-2-related clinical information. A major goal is to use prospective investigation of the Registry to provide interested investigators a rich resource for high priority scientific and clinical questions for the purpose of dissemination of this information through peer reviewed literature. By providing new insights into how to best select appropriate patients and manage them to maximize therapeutic response while limiting toxicity, the PROCLAIM registry will help individualize therapeutic selection for patients with mRCC and mM. The database is a living assessment of modern HD IL-2 use and will provide insight into its evolving use as new therapies emerge. This unique collaboration between physicians, patients, and industry seeks to foster communication, information sharing and innovation regarding HD IL-2’s place in cancer immunotherapy research in a rapidly changing environment.
